# Supplementary material for: Analysis of Electric Field Stimulation in Blue Light Stressed 661W Cells
Source: Int J Mol Sci. 2023 Feb 8;24(4):3433. doi: 10.3390/ijms24043433 (PMC9965974; doi:10.3390/ijms24043433)
Supplement: Supplementary file 1 [file ijms-24-03433-s001.zip › ijms-2175914-supplementary.pdf]

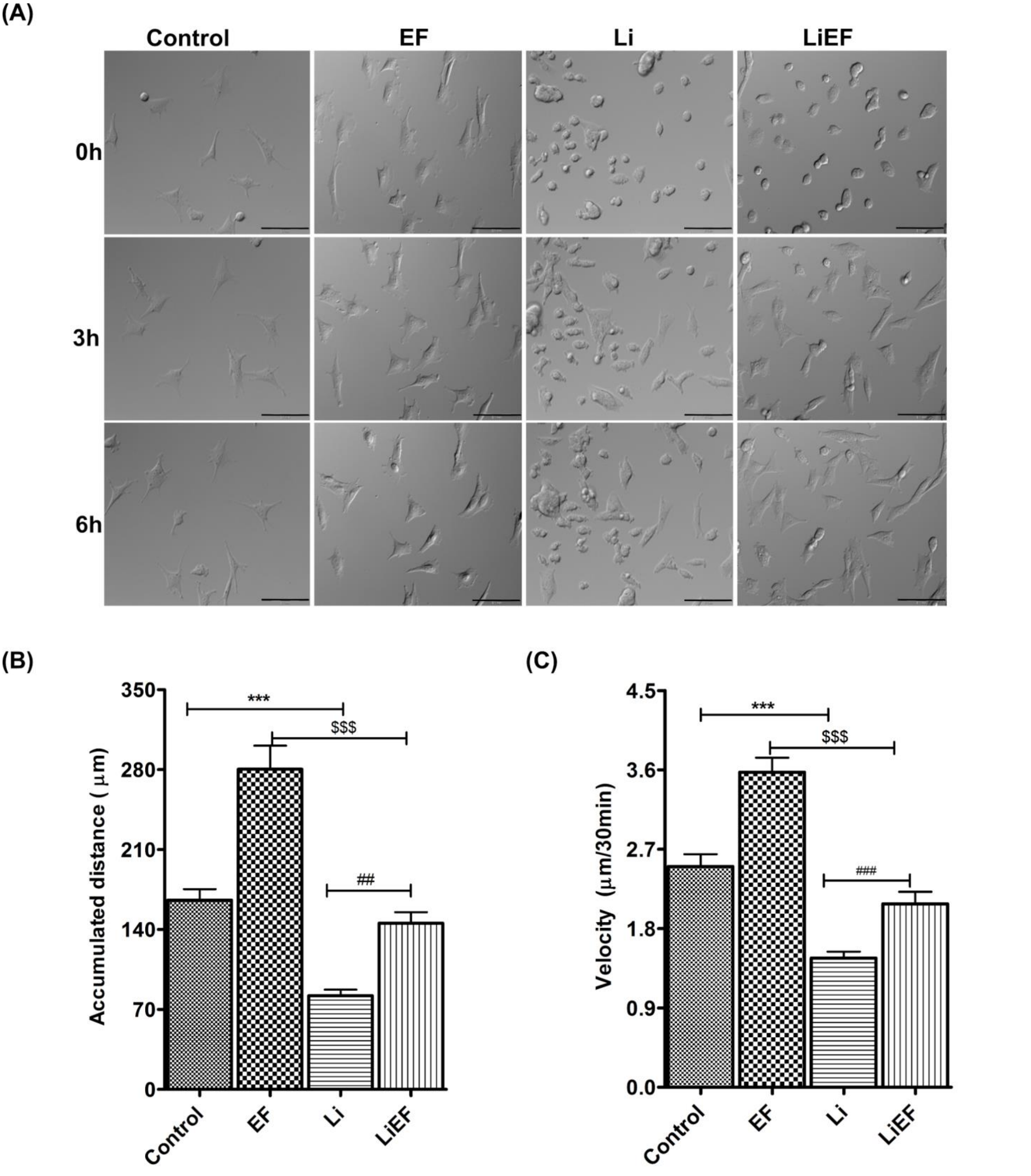

Figure S1. **(A): Morphology of 661W cells during different treated conditions.** Bright field microscopy images of 661W cells after different treatments: control (no Li/no EF); EF (2.5V/cm, 10min); Li (405nm, 1.5mW/cm2, 4h); and LiEF (405nm, 1.5mW/cm2, 4h; 2.5V/cm, 10min). Images were taken at 0h, 3h and 6h post treatment. (Scale bar= 100μm). Li: blue light; EF: direct current electric field; LiEF: Li-treated and EF stimulated. **(B-C): Increased velocity and displacement following EF stimulation.** Images were captured at 0h, 3h, and 6h post treatment at Olympus IX81 inverted microscope (Scale bar= 100μm). Quantification of migratory parameters (**B**) distance travelled and (**C**) migration velocity was calculated by manual cell tracking plugin in Image J along with Ibidi Chemotaxis and migration tool (mean ± S.E.M. of three independent experiments, n=40 cells; ***P<0.001 w.r.t Control; $$$ P<0.001 w.r.t EF; ##P<0.01 w.r.t Li).

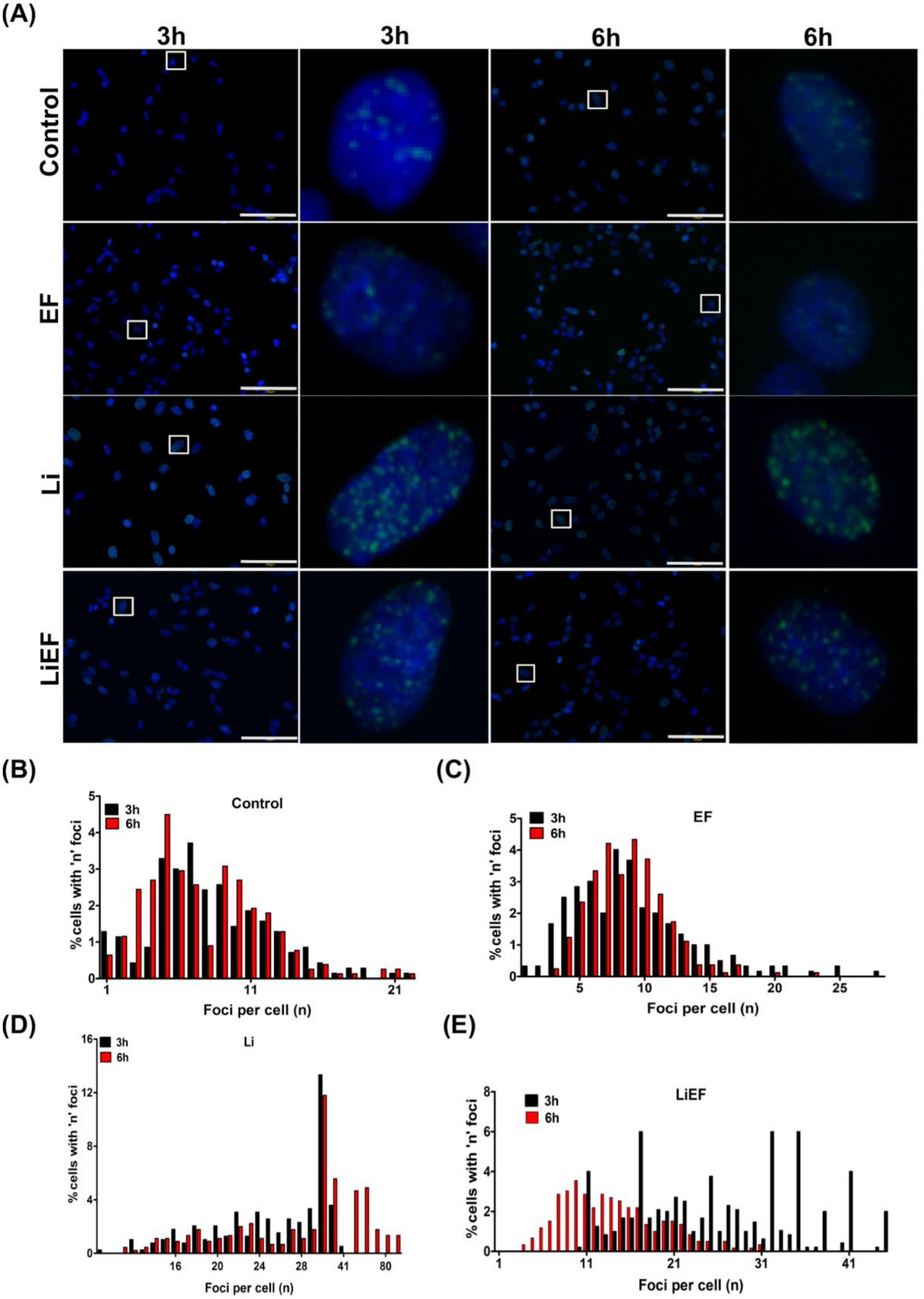

Figure S2. (**A**): **Distribution of γ-H2AX foci in treated 661W cells.** 661W cells were untreated and treated with Li, EF and LiEF and allowed to recover at 37°C for 3h and 6h before fixation and antibody staining. Samples were incubated with H2AX (green) antibody and counter stained with DAPI (blue). The cells shown in (A) are representative images of control, EF, Li, LiEF samples at 3h and 6h. The white boxes in the images indicate the enlarged single cells shown beside. (Scale bar=100μm). Li: blue light; EF: direct current electric field; LiEF: Li-treated and EF stimulated. (**B-E): Quantified percentage of 661W cells with number of γH2AX foci.** 661W cells stained with γH2AX (green) antibody and counter-stained with DAPI (blue) at 3h and 6h. At least 150 cells were counted in triplicates from each time point and the 'n' number of foci per cell was determined using ImageJ counter plugin. Foci per cell (n) were counted at 3h and 6h time point in (**B**) Control, (**C**) EF, (**D**) Li, and (**E**) LiEF. The Y axis represents the percentage of cells with 'n' number of foci per cell nucleus area. The results are mean values of foci number per cell (n). Li: blue light.

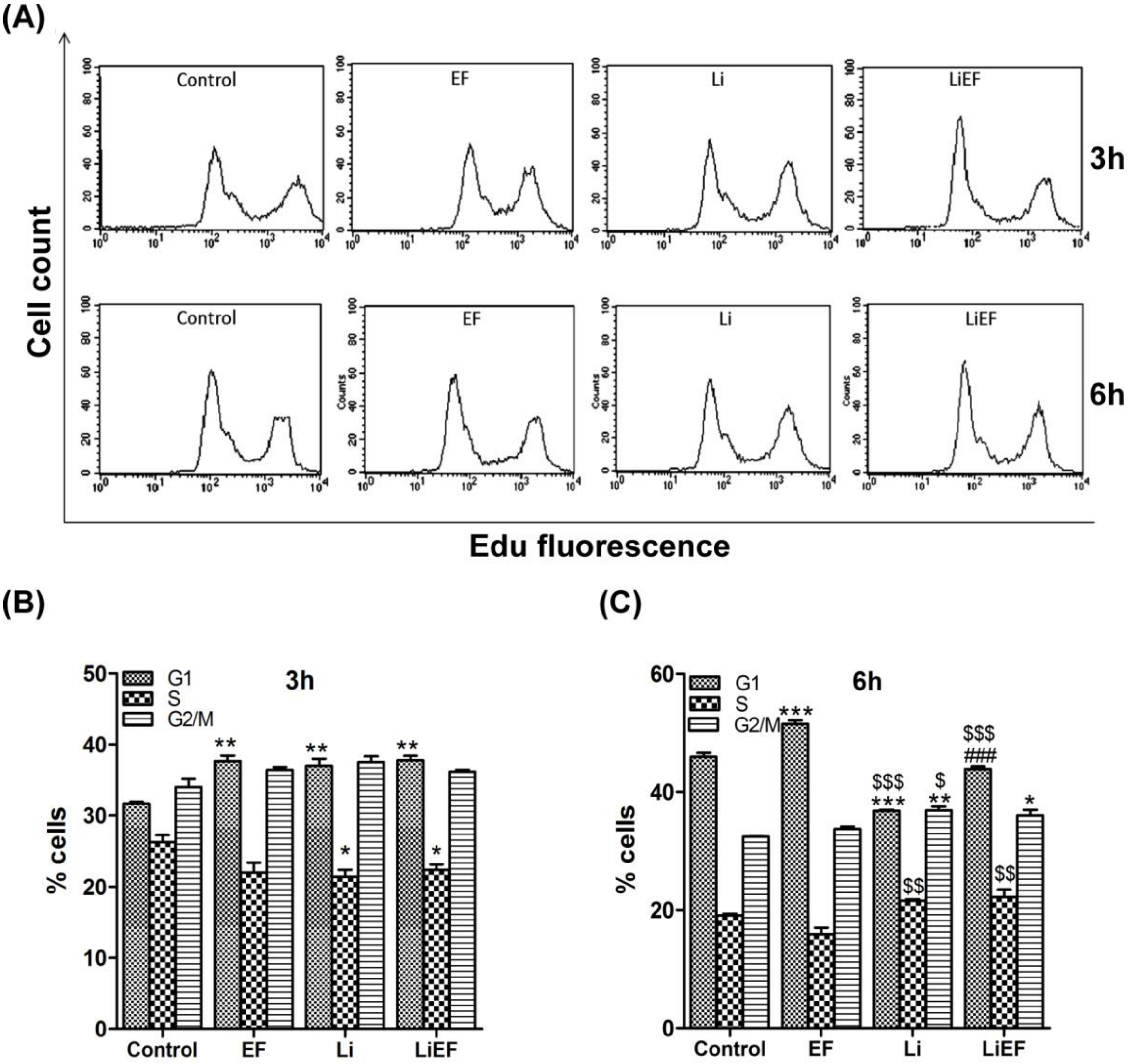

Figure S3. Cell proliferation in treated 661W cells. (**A**): Cell cycle distribution histograms of cells after 3h and 6h following treatment. (**B-C**) Flow cytometric analysis of the percentage of cells in G1, S, or G2/M phases of the cell cycle. (mean + S.E.M.); (n=10000 cells in triplicates; N=3; ***P<0.001, **P<0.01, *P<0.05 w.r.t Control; $$$P<0.001, $$P<0.01, w.r.t EF; ### P<0.001 w.r.t Li). Li: blue light; EF: direct current electric field; LiEF: Li-treated and EF stimulated. s.e.m= standard error mean; n= number of cells; N=number of experiments.

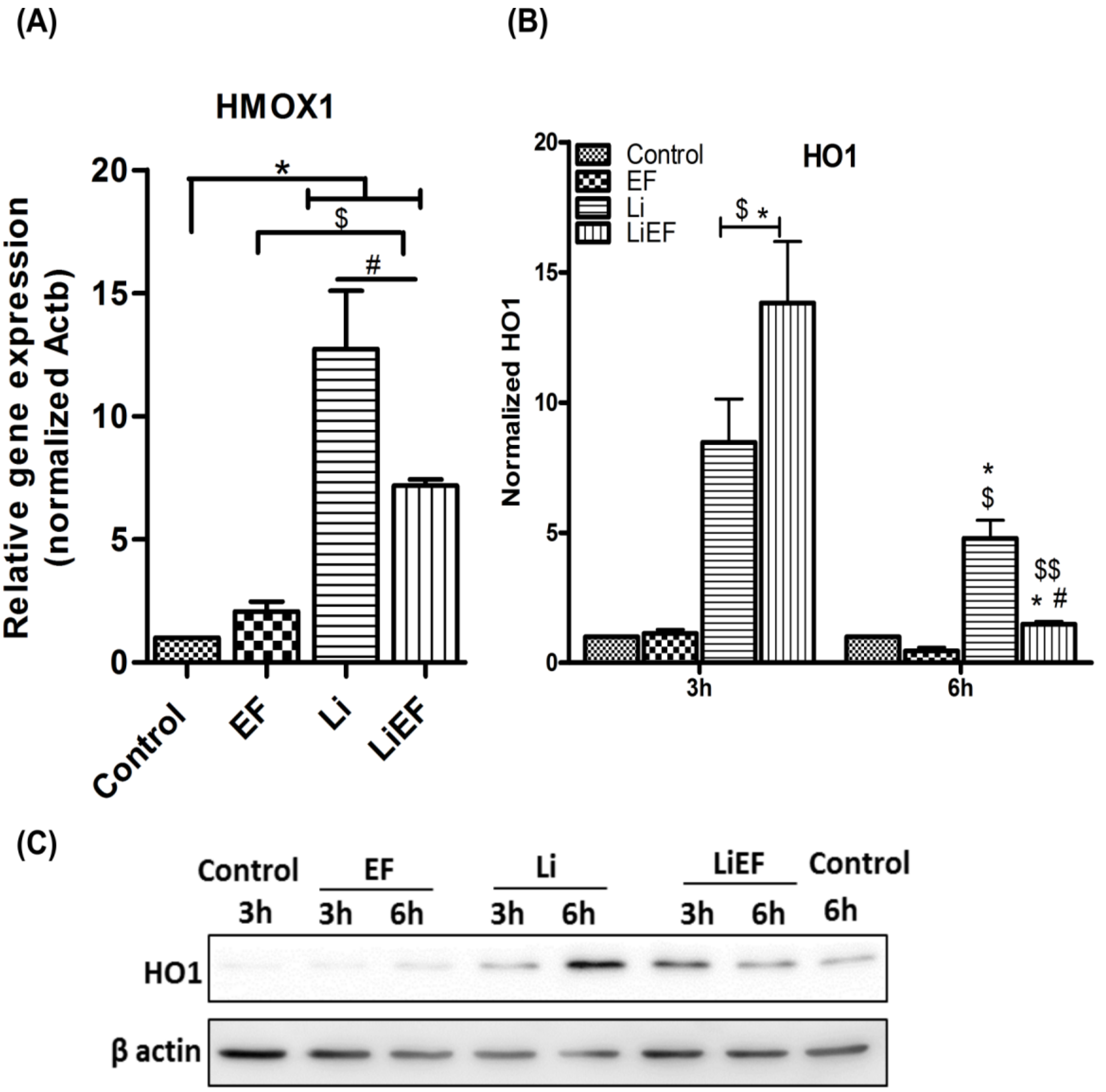

Figure S4. Expression of heme oxygenase-1 levels in treated 661W cells. Heme oxygenase gene and protein levels were measured using qRT-PCR and western blotting methods. (**A**) HMOX1 gene level after 3h post stimulation. The levels were normalized to housekeeping gene β-actin. (**B-C**) Cell lysates were prepared and HO-1 protein expression was measured by western blotting and quantified by using ImageJ (loading control β-actin). Bar graph represents the quantified expression levels of HO-1 at 3h and 6h post treatment. (mean + S.E.M.). (N=3; normalized to control; *P<0.05 w.r.t Control; $$P<0.01, $ P<0.05 w.r.t EF; #P<0.05 w.r.t Li). HMOX1: Heme oxygenase 1; HO1: Heme oxygenase 1; Li: blue light; EF: direct current electric field; LiEF: Li-treated and EF stimulated.

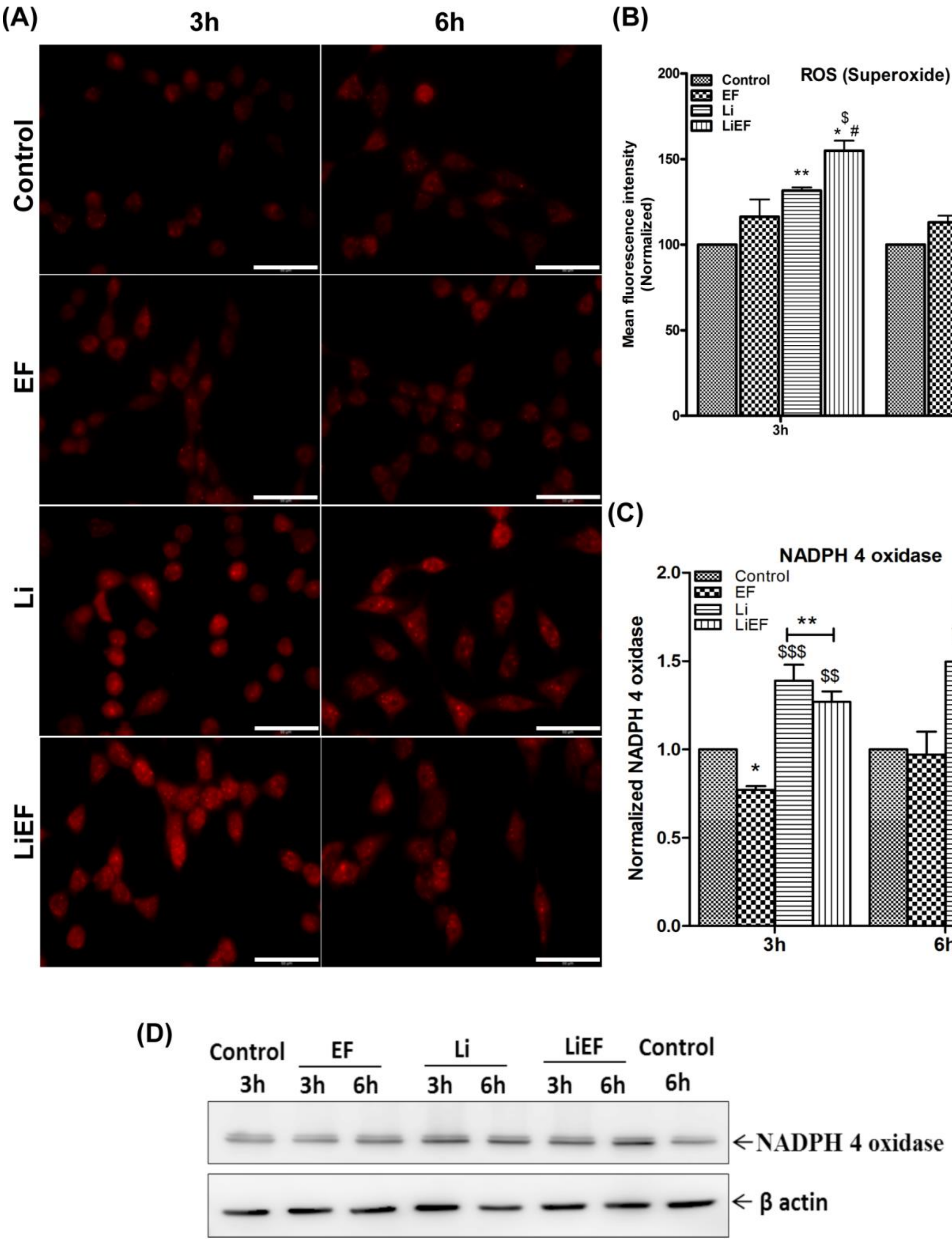

Figure S5. (**A**)**: Superoxide levels in treated 661W cells.** Superoxide levels of cells were measured using DHE dye. (**A**) Staining profile of DHE fluorescence in 661W cells at 3h and 6h following stimulation. DHE: dihydroethidium; Li: blue light; EF: direct current electric field; LiEF: Li-treated and EF stimulated. (Scale bar=20μm). **(B) Bar graph represents DHE fluorescence profile of samples measured using fluorescence flow cytometry at 3h and 6h post treatment (normalized to control).** (mean + S.E.M.). (n=10000 cells in triplicates for flow cytometry; N=3; **P<0.01, *P<0.05 w.r.t control; $ P<0.05 w.r.t EF; # P<0.05 w.r.t Li). DHE: dihydroethidium; Li: blue light; EF: direct current electric field; LiEF: Li-treated and EF stimulated. **(C-D) Expression of NADPH 4 oxidase in treated 661W cells.** Cell lysates were prepared and NADPH 4 oxidase protein expression was measured by western blotting and quantified by using ImageJ. Graph represents quantified protein levels of NADPH 4 oxidase at 3h and 6h post treatment (normalized to control and loading control β actin). (mean±s.e.m). (N=3; **P<0.01, *P<0.05 w.r.t Control, $$$P<0.001, $$P<0.01, $P<0.05 w.r.t EF). Li: blue light; EF: direct current electric field; LiEF: Litreated and EF stimulated.

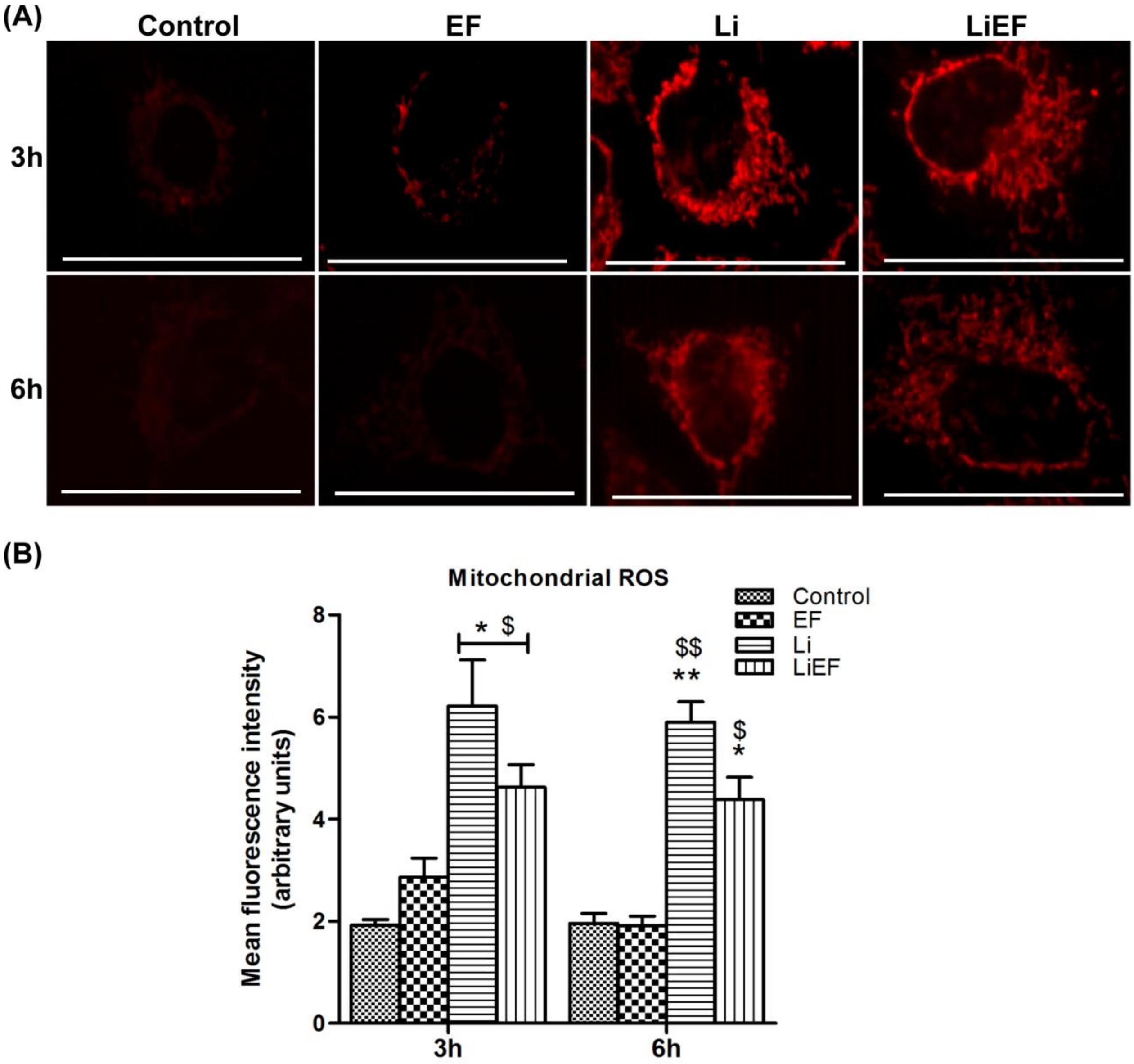

Figure S6. Mitochondrial reactive oxygen species levels in treated 661W cells. (**A**) Fluorescent images of 661W cells stained with MitoSOX red dye to check mitochondrial reactive oxygen species (ROS) levels during different treated conditions (scale bar=50μm). (**B**) The bar graph represents the quantified ROS levels in cells were measured using ImageJ. (mean + S.E.M.). (N=3; **P<0.01, *P<0.05 w.r.t Control; $$P<0.01, $P<0.05 w.r.t EF). Li: blue light; EF: direct current electric field; LiEF: Li-treated and EF stimulated.

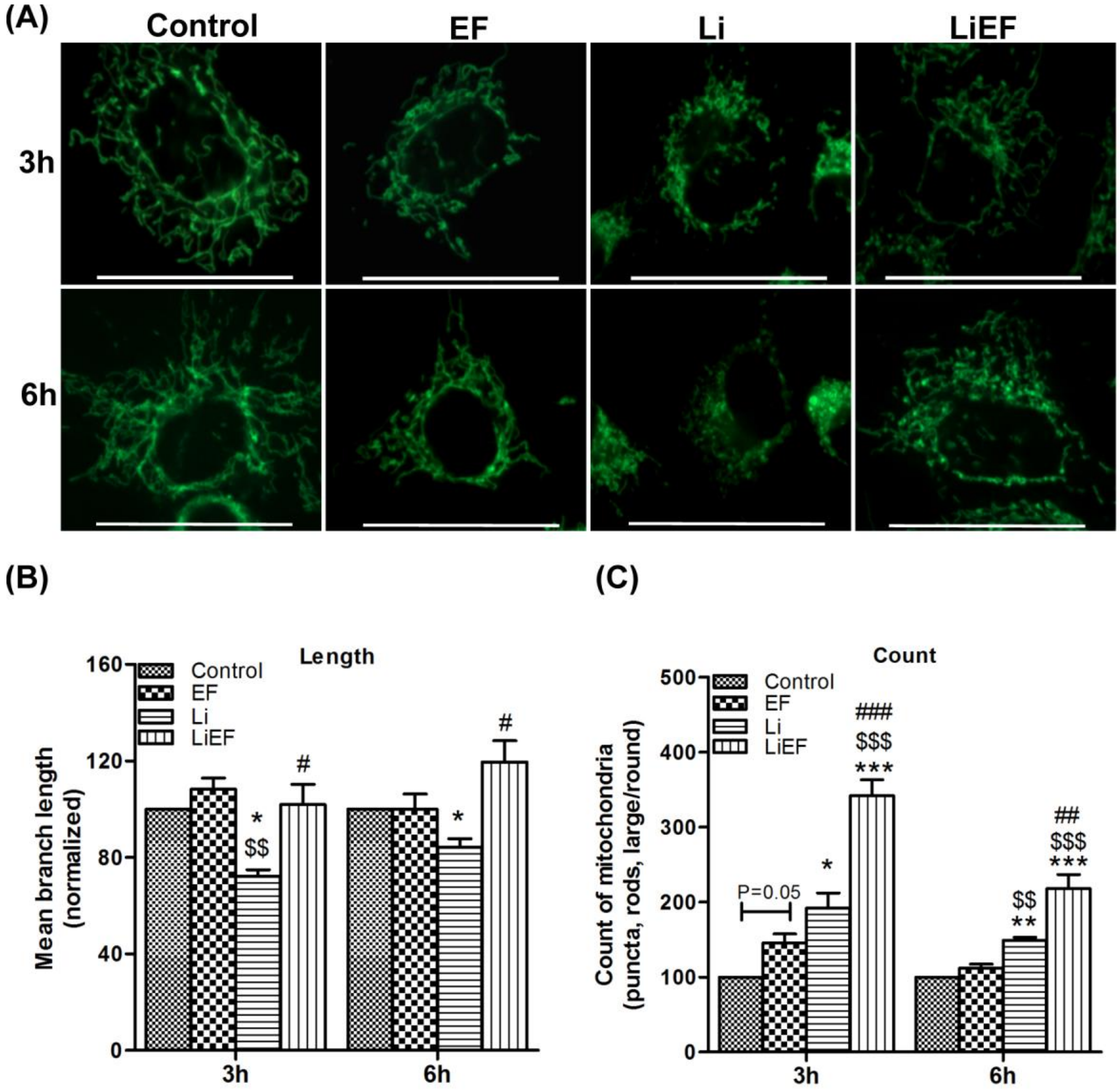

Figure S7. **Mitochondrial morphology in 661W cells during all treated conditions.** (**A**) Fluorescent images of 661W cells stained with MitoTracker green dye to check morphology of mitochondria during different treated conditions (scale bar=50µm). (**B**) The bar graph represents the average length of all branches of mitochondria in cells. (**C**) Bar graph represents the total number of mitochondria either as puncta, rods, and large/round structures represented here as counts. (mean + S.E.M.). (N=3; normalized to control; ***P<0.001, **P<0.01, *P<0.05 w.r.t Control; $$$P<0.001; $$P<0.01 w.r.t EF; ###P<0.001, ##P<0.01, #P<0.05 w.r.t Li). Li: blue light; EF: direct current electric field; LiEF: Li-treated and EF stimulated.

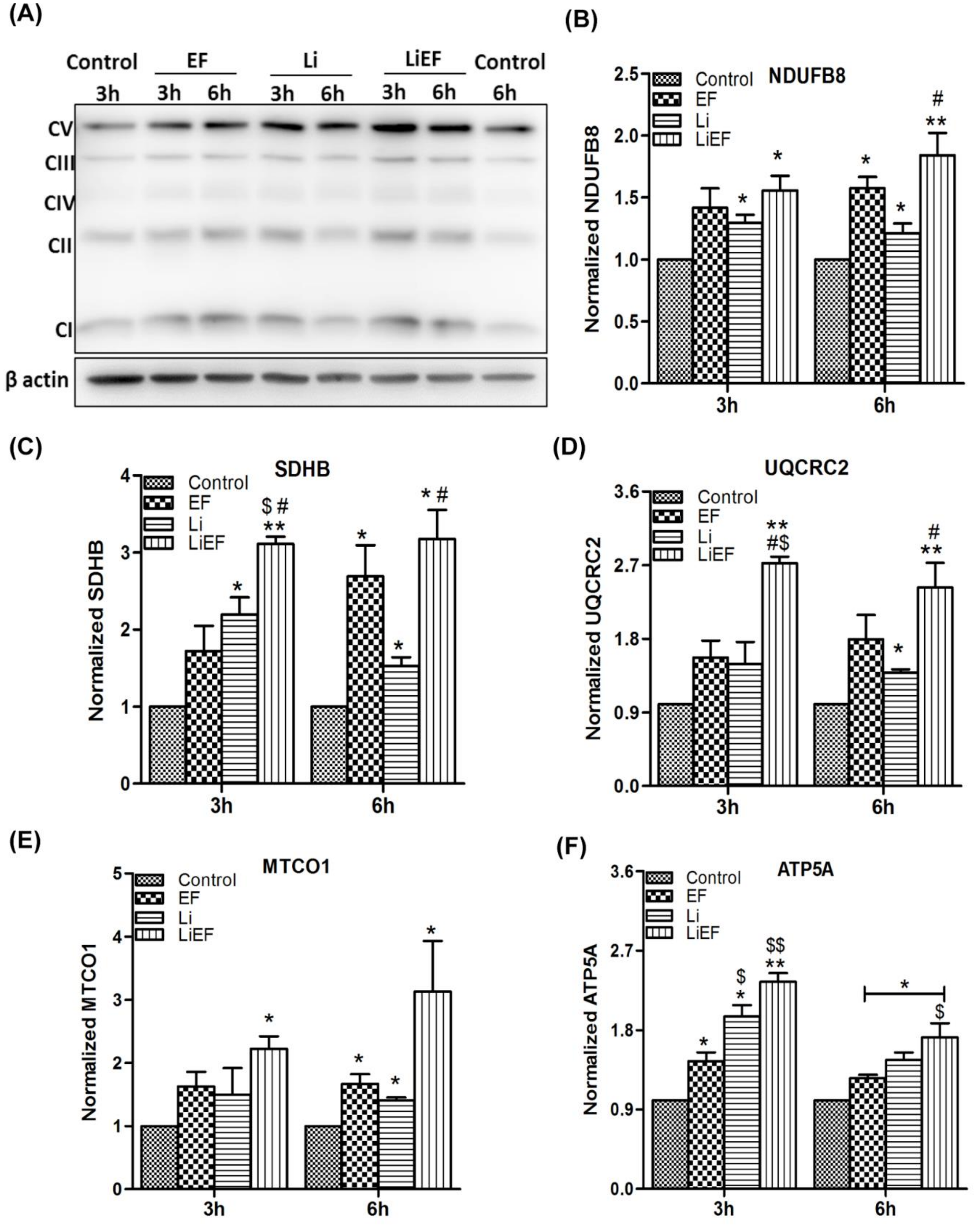

Figure S8. **Quantified levels of mitochondrial complexes in treated 661W cells.**

(**A**) Immunoblotting of cell lysates from differently treated 661W cells using cocktail antibody against OXPHOS proteins: complex I (NDUFB8), complex II (SDHB), complex III (UQCRC2), complex IV (MTCO1), complex V (ATP5A) and loading control β actin. The bar graph represents the quantified band intensity profile of OXPHOS protein: complex I (**B**), complex II (**C**), complex III (**D**), complex IV (**E**) and complex V (**F**) measured using ImageJ. (mean + S.E.M. of three independent experiments; normalized to control; $^{**}P<0.01$, $^{*}P<0.05$ w.r.t Control; \$\$P<0.01 w.r.t EF; \$P<0.05 w.r.t EF; #P<0.05 w.r.t Li). Li: blue light; EF: direct current electric field; LiEF: Li-treated and EF stimulated.

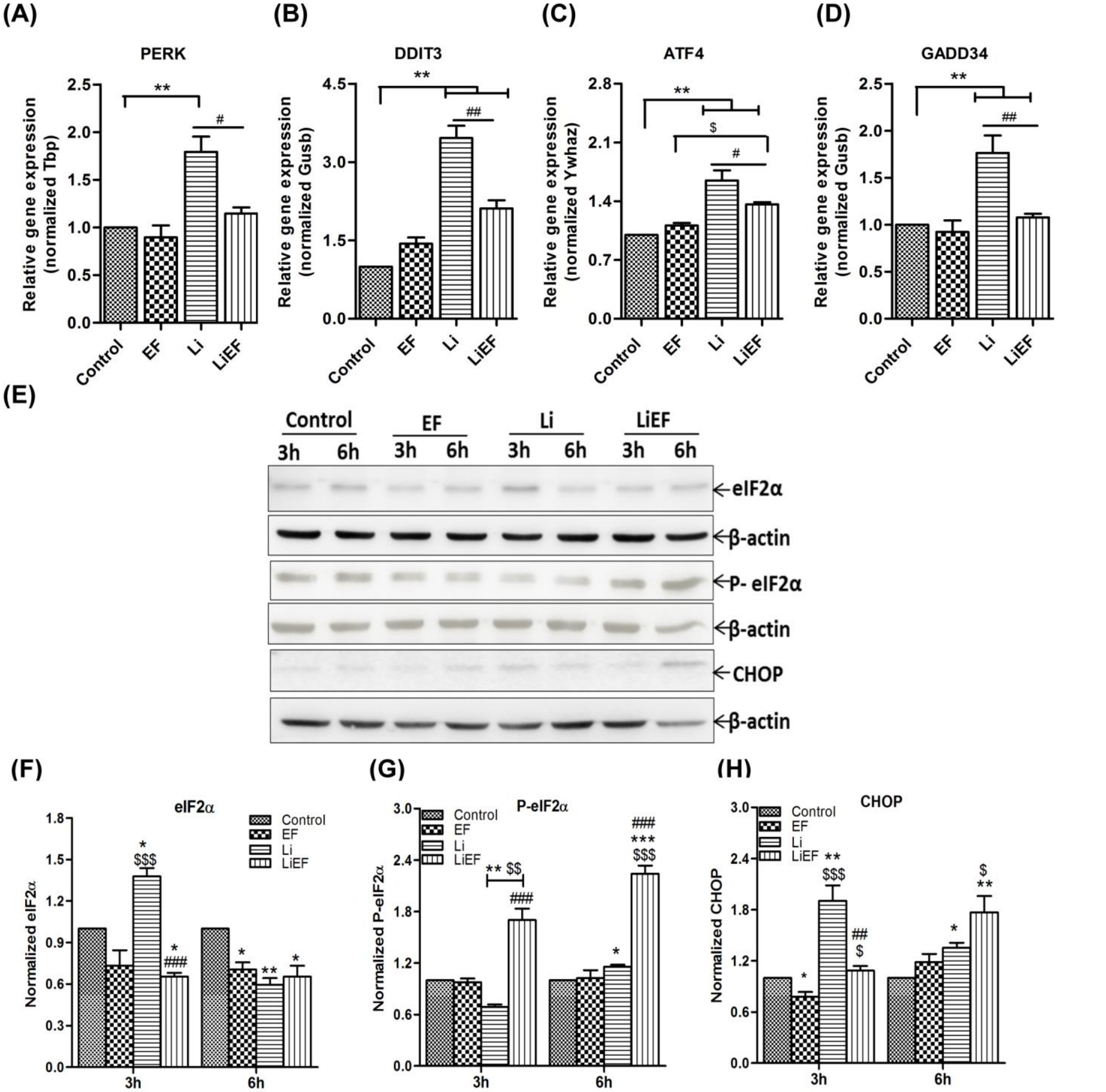

Figure S9. (**A-D) PERK branch-related gene expression in treated 661W cells.** Induction of (**A**) PERK, (**B**) DDIT3, (**C**) ATF4, and (**D**) GADD34 genes at 3h post-treated conditions in 661W cells was quantified using qRT-PCR. Tbp, Ywhaz, Gusb were used as housekeeping genes. Target gene expression relative to housekeeping gene was determined by $2\Delta\Delta ct$ method. (mean + S.E.M.). (N=3; normalized to control; **P<0.01 w.r.t Control; $P<0.05 w.r.t EF; ##P<0.01, #P<0.05 w.r.t Li). PERK: (protein kinase RNA (PKR)-like ER kinase); ATF4: (activating transcription factor-4); GADD34: (growth arrest and DNA damage-inducible protein- 34); DDIT3: (DNA damage-inducible transcript 3); Tbp: (TATA-box binding protein); Ywhaz: (Tyrosine 3-monooxygenase/tryptophan 5-monooxygenase activation protein, zeta polypeptide); Gusb: (beta-glucuronidase); Li: blue light; EF: direct current electric field; LiEF: Li-treated and EF stimulated. s.e.m= standard error mean; N=number of experiments. **(E-H): PERK branch-related protein expression in treated 661W cells.** (**E**) 3h and 6h after Li-exposure and EF stimulation, whole cell lysates were prepared and subjected to western blotting with antibodies against eIF2α, P-eIF2α, and CHOP. β-actin served as loading control. Quantified protein levels of (**F**) eIF2α, (**G**) P-eIF2α, and (**H**) CHOP relative to loading control, β-actin. (mean + S.E.M.). (N=3; normalized to control ; ***P<0.001, **P<0.01, *P<0.05 w.r.t Control; $$$P<0.001, $$P<0.01, $P<0.05 w.r.t EF; ###P<0.001, ##P<0.01, #P<0.05 w.r.t Li). eIF2α: (eukaryotic translation initiation factor-2); PeIF2α: (phosphorylated-eukaryotic translation initiation factor-2); CHOP: (C/EBP-homologous protein); Li: blue light; EF: direct current electric field; LiEF: Li-treated and EF stimulated. s.e.m= standard error mean; N=number of experiments.

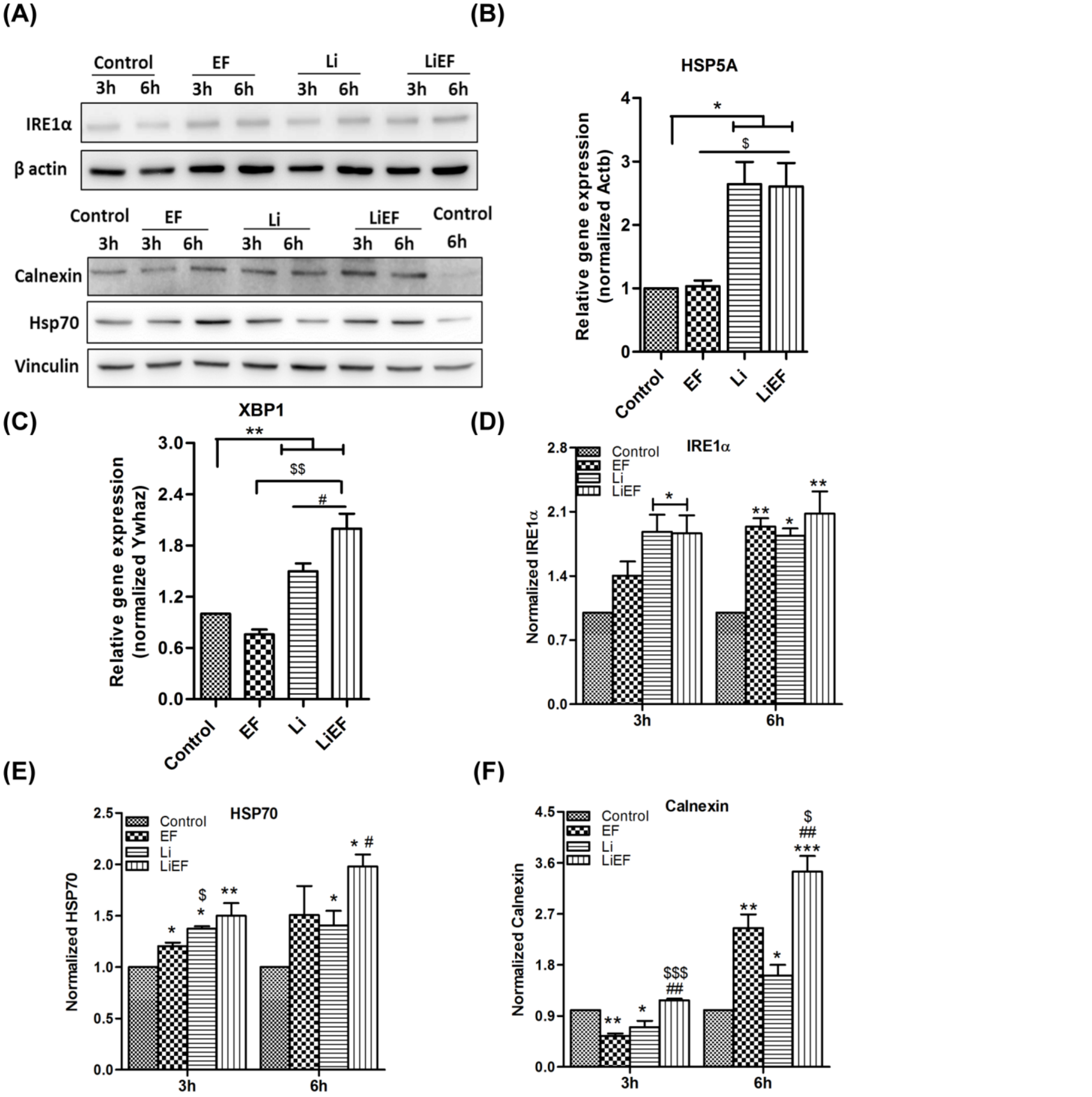

Figure S10. **(A-D) IRE1 branch-related genes and protein expression in treated 661W cells.** Induction of (**B**) HSP5A, and (**C**) XBP1 genes in 661W cells was quantified using qRT-PCR. Ywhaz and Actb were used as housekeeping genes. Target gene expression relative to housekeeping gene was determined by 2ΔΔct method. (**A, D**) Whole cell lysates were prepared and subjected to western blotting with antibody against IRE1α. β-actin served as loading control. Quantified protein levels of IRE1α relative to loading control. (mean + S.E.M.). (N=3; normalized to control; **P<0.01, *P<0.05 w.r.t Control; $$P<0.01, $P<0.05 w.r.t EF; #P<0.05 w.r.t Li). HSPA5: (Heat Shock Protein Family A (Hsp70) Member 5); IRE1α: (inositol-requiring protein-1); XBP1: (X-box binding protein-1); Li: blue light; EF: direct current electric field; LiEF: Li-treated and EF stimulated. S.E.M. = standard error mean; N=number of experiments. **(A,E-F): Chaperone protein expression levels in treated 661W cells.** Whole cell lysates were prepared and subjected to western blotting with antibody against (**A**) HSP70 and calnexin. Vinculin served as loading control. Quantified protein levels of HSP70 (**E**) and calnexin (**F**) relative to loading control. (mean + S.E.M.). (N=3; normalized to control; ***P<0.001, **P<0.01, *P<0.05 w.r.t Control; $$$P<0.001, $P<0.05 w.r.t EF; ##P<0.05, #P<0.05 w.r.t Li). HSP70: (Heat Shock Protein 70); Li: blue light; EF: direct current electric field; LiEF: Li-treated and EF stimulated; N=number of experiments.

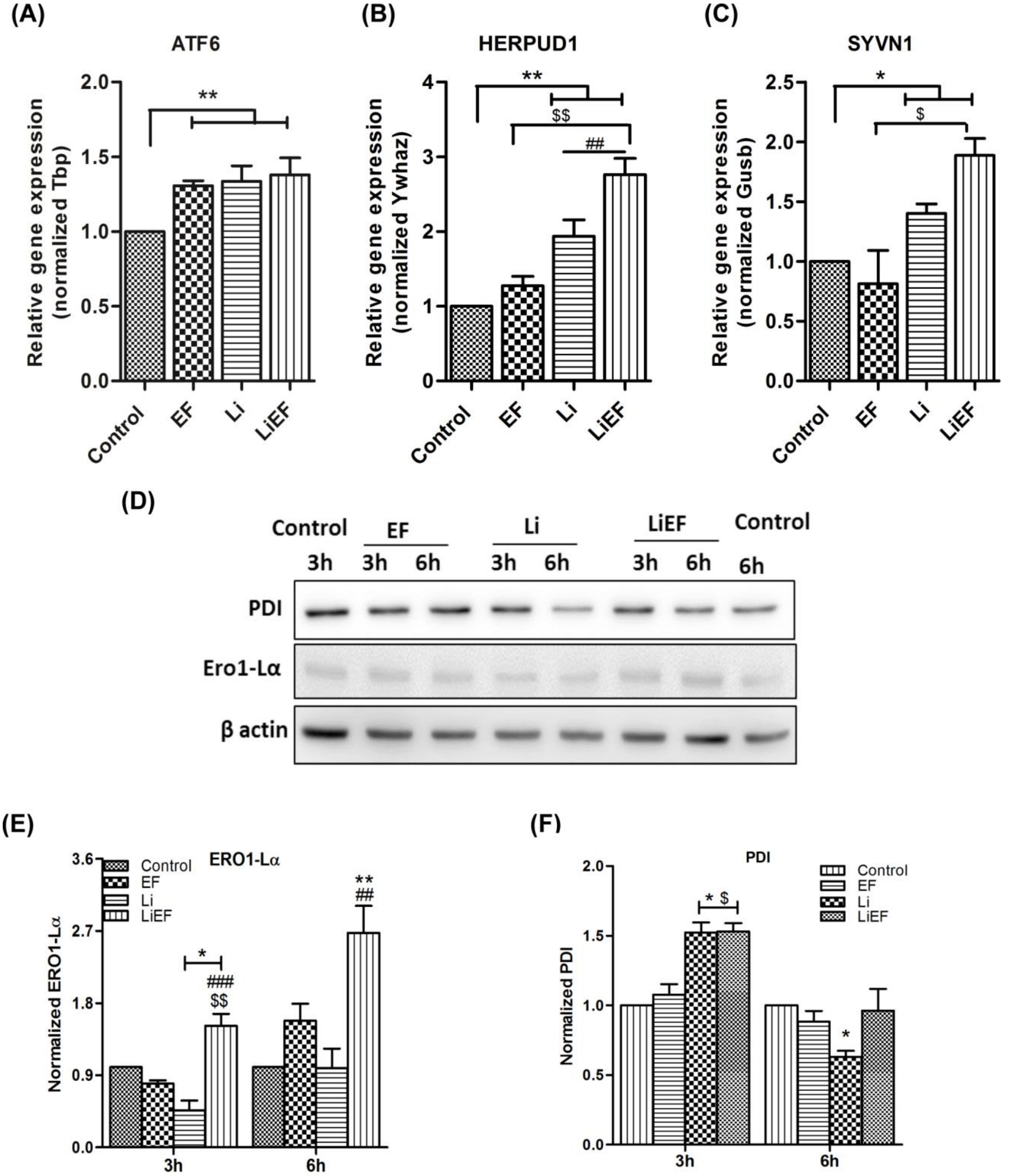

Figure S11. **(A-C): ATF6 branch-related gene expression in treated 661W cells.**
Induction of (**A**) AT6, (**B**) HERPUD1, and (**C**) SYVN1 genes at 3h post treated conditions in 661W cells was quantified using qRT-PCR. Tbp, Ywhaz, Gusb were used as housekeeping genes. Target gene expression relative to housekeeping gene was determined by 2ΔΔct method. (mean + S.E.M.). (N=3; normalized to control; **P<0.01, *P<0.05 w.r.t Control; $$P<0.01, $P<0.05 w.r.t EF; ##P<0.01 w.r.t Li). ATF6: (protein kinase RNA (PKR)-like ER kinase); HERPUD1: (activating transcription factor-4); SYVN1: (growth arrest and DNA damage inducible protein-34); Ywhaz: (Tyrosine 3-monooxygenase/tryptophan 5-monooxygenase activation protein, zeta polypeptide); Gusb: (beta-glucuronidase); Li: blue light; EF: direct current electric field; LiEF: Li-treated and EF stimulated. s.e.m= standard error mean; N=number of experiments. (**D-F): Protein expression of ERO1-Lα and PDI in treated 661W cells.** (**D**) 3h and 6h after Li-exposure and EF stimulation, whole cell lysates were prepared and subjected to western blotting with antibodies against ERO1-Lα and PDI.. β-actin served as loading control. Quantified protein levels of (**E**) ERO1-Lα and (**F**) PDI relative to loading control, β-actin. (mean + S.E.M.). (N=3; normalized to control ; **P<0.01, *P<0.05 w.r.t Control; $$P<0.01, $P<0.05 w.r.t EF; ###P<0.001, ##P<0.01 w.r.t Li). ERO1-Lα: (endoplasmic reticulum oxidoreductin 1-Lα): PDI: (protein disulphide-isomerase); Li: blue light; EF: direct current electric field; LiEF: Li-treated and EF stimulated.

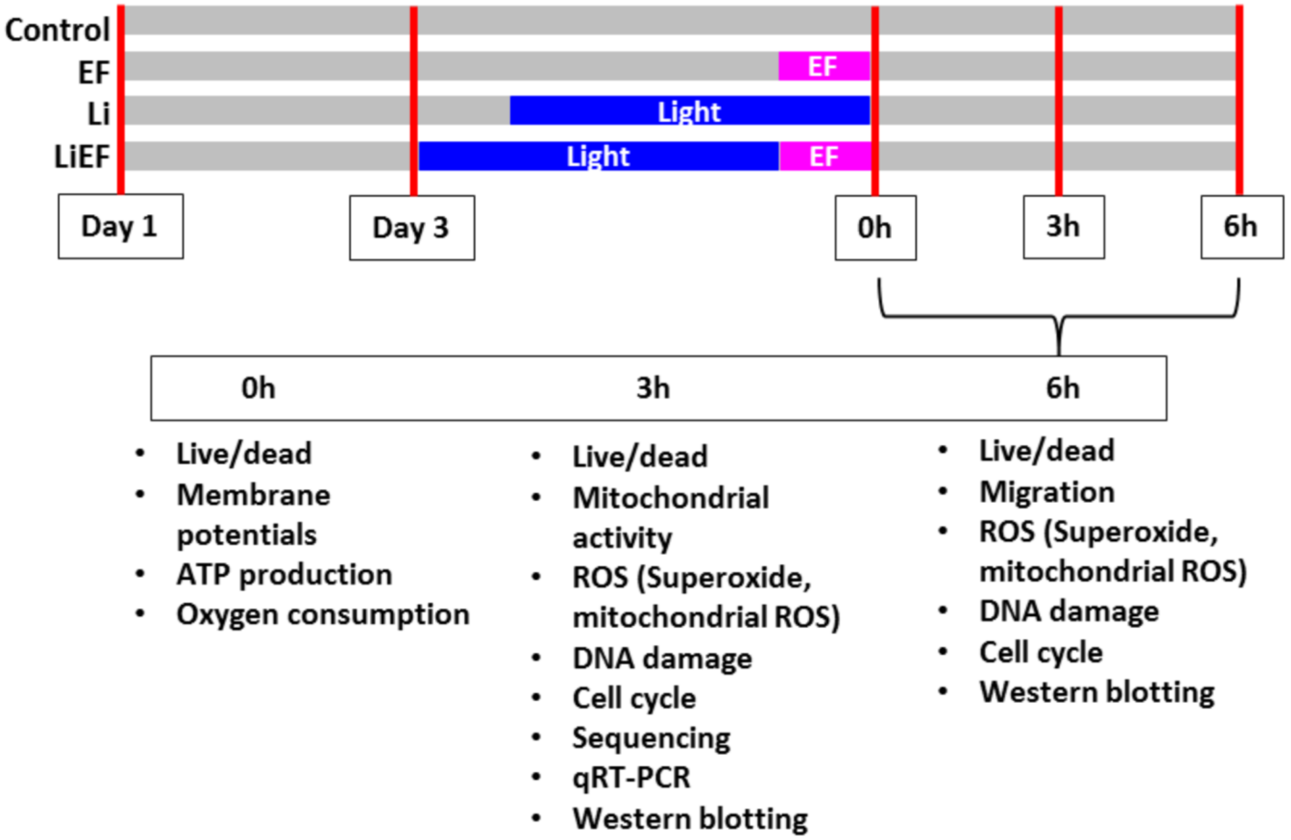

Figure S12. **Schematic representation of established treatment method for 661W cells.** EF: direct current electric field; 0h: 0 hours; 3h: 3 hours; 6h: 6 hours; ATP: Adenosine triphosphate; ROS: Reactive oxygen species; DNA: Deoxyribonucleic acid; qRT-PCR: Quantitative reverse transcription-polymerase chain reaction;
